# Hepatic mitochondrial bioenergetics and metabolism across lactation and in response to heat stress in dairy cows*

**DOI:** 10.3168/jdsc.2023-0432

**Published:** 2023-11-17

**Authors:** Amy L. Skibiel

**Affiliations:** Department of Animal, Veterinary and Food Sciences, University of Idaho, Moscow, ID 83844

## Abstract

Lactation is energetically demanding for the dairy cow. Numerous morphological and metabolic changes orchestrated across different tissues in the body partition nutrients for milk synthesis. The liver is a key organ coordinating modifications in metabolism that increase substrate availability for the mammary gland. Impaired capacity to make the needed physiological adjustments for lactation, such as occurs with heat stress, can result in metabolic disease and poor lactation performance. At the cellular level, increases in mitochondrial density and bioenergetic and biosynthetic capacity are critical adaptations for successful lactation, providing energy and substrates for milk synthesis. Mitochondria are also involved in coordinating adaptation to a variety of stressors by providing the metabolic foundation to enlist a stress response. Heat stress can damage mitochondrial structures and impair mitochondrial function, with implications for pathogenesis and production. This systematic review focuses on the hepatic mitochondrial adaptations to lactation and the mitochondrial responses to heat stress. Future research directions are also discussed that may lead to improvements in managing the metabolic needs of the lactating cow and diminishing the adverse production and health consequences from environmental stress.

## Graphical Abstract

**Figure:**
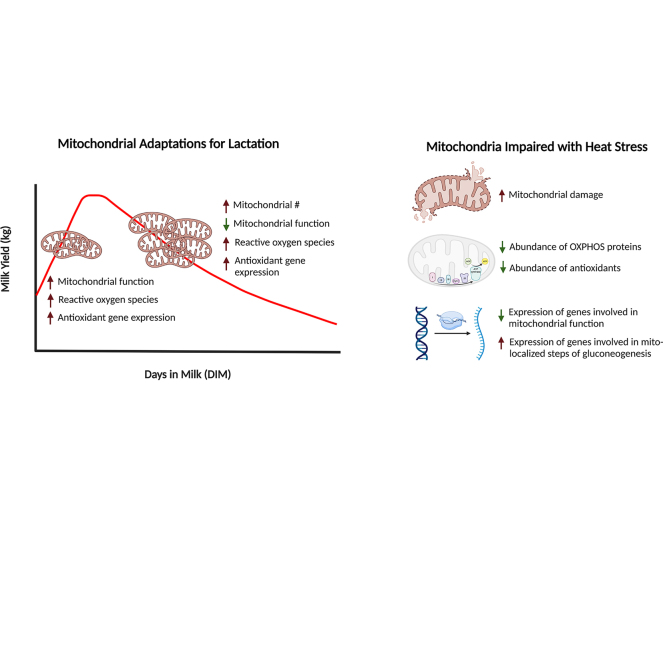

Lactation is the most energetically challenging life stage for female mammals (Gittleman and Thompson, 1988). This is particularly apparent in the modern-day dairy cow, which produces more than 10,000 kg of milk in a single lactation, requiring up to 7 times more energy than the maintenance energy requirement (Baumgard et al., 2017). Homeorhetic adjustments in tissue-specific and systemic metabolism are necessary to provide the nutrients and energy needed for milk synthesis. The liver is an important organ orchestrating metabolic adaptations for lactation by increasing the production of alternative energy fuels and increasing glucose availability, ensuring a steady supply of nutrients for the mammary gland (Reynolds et al., 2003). Impaired ability to enlist physiological adjustments for lactation can lead to metabolic disorders and decreased milk production (Drackley, 1999).

Elevated ambient temperature and humidity are environmental conditions that compromise propensity for physiological adaptation to lactation in dairy cows. Dairy cows are particularly sensitive to thermal stress as high metabolic rates associated with milk production generate a substantial amount of heat (Kadzere et al., 2002). Physiological mechanisms are employed in the heat-stressed dairy cow that prioritize thermoregulation over milk production, resulting in markedly altered macronutrient metabolism and depressed milk synthesis (Rhoads et al., 2009; Wheelock et al., 2010). For example, reduced lipid mobilization from adipose tissue and greater peripheral reliance on glucose for energy are characteristic metabolic alterations in heat-stressed dairy cattle (Baumgard and Rhoads, 2013).

Mitochondria play crucial roles in cellular bioenergetics and biosynthesis and regulate the energetic response to stress (Picard et al., 2018). The importance of mitochondrial function in lactation has long been appreciated and altered mitochondrial structure and function have been implicated in the aberrant metabolic state of heat-stressed cows. Coordinated changes in various aspects of mitochondrial behavior across mammary and extramammary tissues are necessary to support the nutrient and energy requirements of milk synthesis (Mowry et al., 2017). During heat stress, mitochondria can become swollen with broken membranes, such as occurs in rat skeletal muscle, which compromises organelle integrity (Hsu et al., 1995). Heat stress also induces mitochondrial dysfunction that contributes to cellular damage and may divert resources away from the mammary gland, which has consequences for cattle health and performance (Belhadj Slimen et al., 2016; Marquez-Acevedo et al., 2023b).

This systematic review discusses the current body of knowledge regarding mitochondrial adaptations to lactation and to heat stress in dairy cows. Although the focus of this review is on liver mitochondrial metabolism, comparisons across tissues are made where relevant. Gaps in knowledge and areas warranting further exploration are also highlighted.

The onset of lactation is associated with an increase in the nutrient and energy requirements of the cow. Compared with several decades ago, the proportion of ME requirement used for milk production has doubled in the modern dairy cow, with 65% of ME being used for milk synthesis (Baumgard et al., 2017). Several homeorhetic adjustments, including greater feed consumption, mobilization of tissue nutrient reserves, and changes in nutrient partitioning, divert needed resources to the mammary gland to support milk synthesis (Bauman and Currie, 1980). Although feed intake increases in the dairy cow in early lactation, intake alone is insufficient to meet the demands of maintenance and lactation, causing a negative energy balance in the cow (Drackley et al., 2005). This adaptive feature of lactation in dairy cows induces physiological and metabolic changes across mammary and extramammary tissues (e.g., liver, adipose, skeletal muscle) that partition resources for milk synthesis and increase mammary nutrient uptake and use. As a result, milk yield continues to increase to peak lactation despite the negative energy balance.

The liver is a key organ orchestrating adaptive changes in macronutrient metabolism at the onset of lactation. Increased hepatic gluconeogenesis and glycogenolysis generate glucose that is predominantly used to support milk lactose synthesis in the mammary gland (Drackley et al., 2005). Nonesterified fatty acids (**NEFA**) undergo β-oxidation in the mitochondria of hepatocytes, producing NADH and FADH_2_, and thus are indirectly used to produce ATP through oxidative phosphorylation (**OXPHOS**; Reynolds et al., 2003). In addition, NEFA are partially oxidized in the liver, yielding ketone bodies that are used for milk fat synthesis and as an alternate energy source for peripheral tissues, thereby sparing glucose for milk synthesis in the glucose-limited lactating ruminant (Drackley et al., 2005). Amino acids taken up by the liver can also be used for ATP production and, to a lesser extent than propionate, serve as substrates for gluconeogenesis (Schäff et al., 2012).

Mitochondria produce more than 90% of the cellular energy (i.e., ATP) in eukaryotic cells through OXPHOS (Lane and Martin, 2010). This process occurs through the electron transport chain (**ETC**), consisting of 4 enzyme complexes (complex I through IV) spanning the inner mitochondrial membrane. Reducing equivalents (i.e., NADH and FADH_2_) produced in the mitochondrial TCA cycle and through mitochondrial β-oxidation are oxidized and their electrons transferred through the ETC through a series of reduction-oxidation reactions. NADH initially donates electrons to complex I and FADH_2_ to complex II. As electrons are transferred across successive complexes in the ETC, protons are pumped out of the mitochondrial matrix generating an electrochemical proton gradient across the inner mitochondrial membrane that drives the phosphorylation of ADP through the enzymatic activity of ATP synthase (Lodish et al., 2003).

Various mitochondrial properties change dynamically according to the energy needs of the cell. Generally, mitochondrial density and OXPHOS increase when energy needs rise (Nunnari and Suomalainen, 2012). With the exception of a study in dairy cows showing a higher number of liver mitochondria in pregnant cows in late lactation relative to early lactation (Laubenthal et al., 2016), little is known about hepatic mitochondrial physiology during lactation in cattle. However, our group recently conducted a study to assess functional changes in hepatic mitochondria across lactation and in association with milk yield (Favorit et al., 2021). Liver biopsies were collected from a group of Holstein dairy cows at early (8 DIM), peak (75 DIM), and late (199 DIM) lactation. Late-lactation cows were also pregnant. Mitochondrial oxygen consumption was measured after providing either NADH-linked (e.g., pyruvate, malate, glutamate) or FADH_2_-linked (e.g., succinate) substrates and ADP to isolated mitochondria. Respiratory control ratio (**RCR**) was calculated as the state 3 (maximal, ADP-stimulated) to state 4 (resting) respiration rates. We found that NADH-linked RCR did not change across lactation; however, FADH_2_-linked RCR increased in early lactation, when NEFA concentrations were highest, and RCR decreased from peak to late lactation (Figure 1). Mitochondrial FADH_2_-linked respiration was also positively associated with milk yield (Favorit et al., 2021). Together, our results indicate greater coupling of substrate oxidation to ATP production and increased use of NEFA as substrates for OXPHOS in the liver of dairy cows in early lactation when milk yield and energy demands are highest.

**Figure 1:**
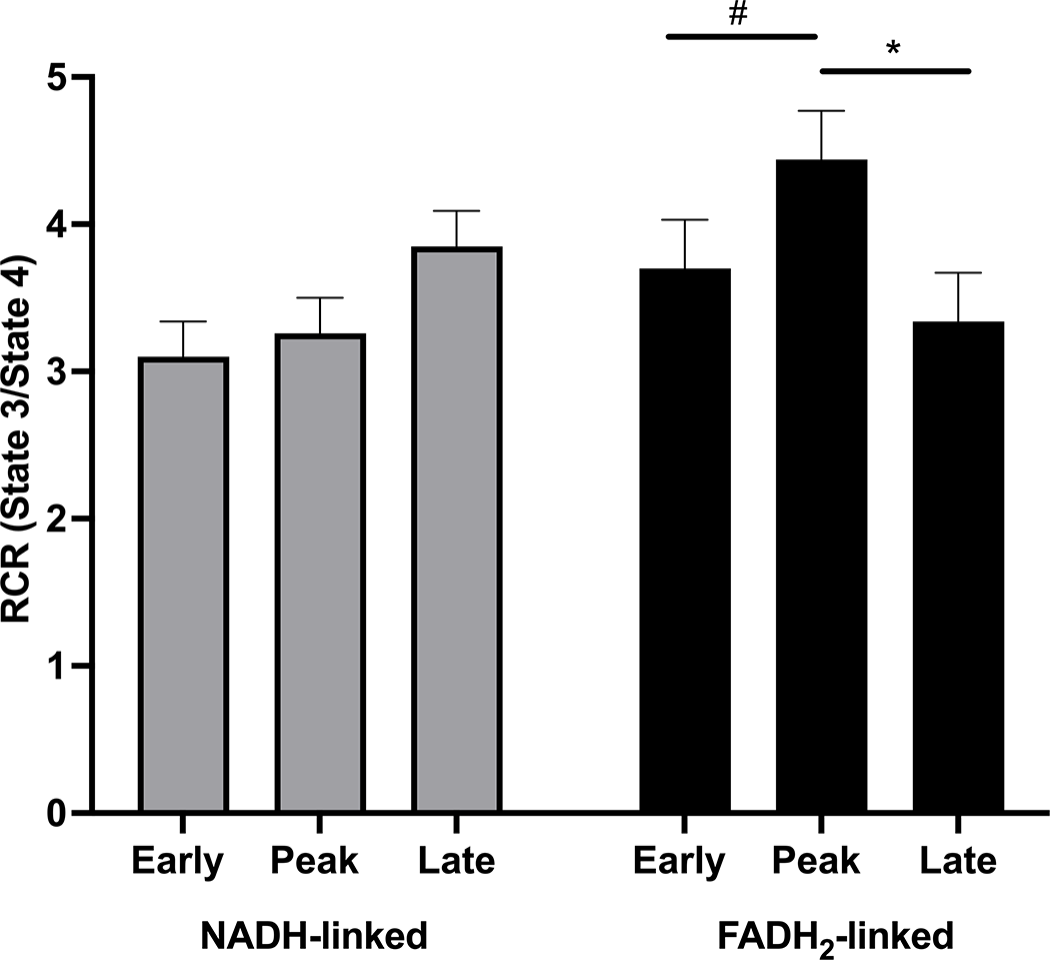
Mitochondrial respiratory control ratio (RCR) of liver tissue using NADH-linked (gray bars) or flavin adenine dinucleotide (FADH_2_)-linked (black bars) substrates. Liver biopsies were harvested from 11 multiparous Holstein cows at 8 ± 2, 75 ± 4, and 199 ± 6 DIM (± SE), encompassing early, peak, and late lactation, respectively. #*P* < 0.1, *P < 0.05. Data from Favorit et al. (2021). Error bars are SE.

At the molecular level, mitochondrial biogenesis and bioenergetics are mediated by nuclear receptors and transcription activators that regulate metabolic homeostasis and energy production (Gutgesell et al., 2009). Peroxisome proliferator-activated receptor-gamma coactivator 1-α (PGC1α; gene symbol *PPARGC1A*) is a transcriptional coactivator that responds to cellular energy need, through activation by AMP-activated protein kinase (AMPK) and other enzymes, and enhances PPAR-mediated gene transcription (Cagin and Enriquez, 2015). Peroxisome proliferator-activated receptors (PPARα, PPARδ, PPARγ; gene symbols *PPARA*, *PPARD*, *PPARG*, respectively), a family of nuclear transcription factors, modulate the transcription of genes involved in lipid uptake, fatty acid (**FA**) oxidation, and glucose metabolism (Gutgesell et al., 2009; Bionaz et al., 2013). In addition to PPAR, nuclear respiratory factors (NRF-1 and NRF-2) are targets of PGC1α, thereby upregulating transcription of nuclear-encoded mitochondrial transcription factors (TFAM, TFBM) that control mitochondrial DNA (**mtDNA**) maintenance, replication, and transcription (Virbasius and Scarpulla, 1994). The NRF also control mitochondrial oxidative capacity and function by directly modulating expression of nuclear genes encoding protein subunits of the ETC (Scarpulla, 2012). Sirtuins (**SIRT**) are another group of regulatory molecules, specifically histone deacetylases, that play a role in modulating mitochondrial metabolism in accordance with cellular energy demand. For example, the nuclear-localized SIRT1 activates PGC1α when cellular NADH concentration drops to increase mitochondrial biogenesis, gluconeogenesis, and FA oxidation (Chalkiadaki and Guarente, 2012; Ghinis-Hozumi et al., 2013). Activity of mitochondrial SIRT3 is also enhanced by low cellular NADH levels, resulting in activation of metabolic enzymes involved in OXPHOS, FA oxidation, and ketogenesis (Shih and Donmez, 2013). In addition, SIRT3 has been shown to activate antioxidants, attenuating risk of cellular oxidative stress (Chalkiadaki and Guarente, 2012).

Few studies have characterized the molecular pathways involved in mitochondrial adaptation to lactation. In dairy cows, *PPARGC1A* expression in the liver and mammary gland increased from late pregnancy to early lactation, to promote mitochondrial biogenesis and mobilization/uptake of substrates for energy and milk production (Wang et al., 2015). However, across lactation, results are equivocal, with one study reporting a decrease in hepatic *PPARGC1A* expression from early to late lactation (Laubenthal et al., 2016) and another finding no change in *PPARGC1A* expression across lactation (Favorit et al., 2021). Our group also found that expression of *NRF1*, *TFAM*, and *TFBM* increased in liver tissue of dairy cows from early lactation to late lactation despite the similar expression of *PPARGC1A* across lactation (Favorit et al., 2021). In contrast, Laubenthal et al. (2016) did not observe changes in expression of *NRF1* or *TFAM* across lactation even though *PPARGC1A* was downregulated. Discrepancies among studies may reflect pregnancy-, parity-, and productive stage-dependent alterations in mito-nuclear signaling. For example, in the Laubenthal et al. (2016) study, late-lactation samples were collected more than 100 d later than the late-lactation samples in Favorit et al. (2021).

In dairy cattle, other components of metabolic signaling pathways are upregulated in the liver during the transition period, coinciding with elevated NEFA, such as *PPARA* (Loor et al., 2005; Wang et al., 2015), which regulates FA uptake and oxidation, and *PPARD* (Favorit et al., 2021), involved in FA catabolism and glucose metabolism, although the latter function in ruminants is not clear (Gutgesell et al., 2009; Bionaz et al., 2013; Hassan et al., 2021). Similar changes in *PPARA* have been reported with nutrient restriction in dairy cows (Loor et al., 2007). Upregulation of *PPARD* in early lactation of dairy cows may promote FA oxidation and possibly plays a role in hepatic glucose metabolism, although this has yet to be studied in the ruminant. Nevertheless, in dairy cows, although the PGC1α/PPAR pathway appears critical to support liver energy metabolism during lactation, it is possible that only specific components of the pathway are upregulated, rather than the entire pathway (Bionaz and Loor, 2012). Moreover, it is important to note that many factors in these signaling pathways are modified post-transcriptionally or post-translationally, so changes in gene expression may not necessarily correlate to functional effects (Fernandez-Marcos and Auwerx, 2011). Further research is needed to fully elucidate the signaling pathways modulating homeorhetic adjustments in mitochondrial metabolism across lactation in the dairy cow.

Heat stress induces a suite of adaptive behavioral and physiological changes in dairy cattle that prioritize thermoregulation and survival. For example, reduced DMI and milk production are hallmarks of heat stress in dairy cattle, functioning to curb the heat increment of rumination, digestion, and milk production (Kadzere et al., 2002). Thermally stressed cows exhibit altered postabsorptive metabolism. Relative to pair-fed thermoneutral cows, lactating heat-stressed cows have lower circulating NEFA and BHB, which are typically elevated in early lactation, greater circulating insulin concentration, and a reduced NEFA response to an epinephrine challenge (Rhoads et al., 2009; Baumgard et al., 2011). Under thermoneutral conditions, epinephrine, a catabolic hormone, along with reduced circulating insulin, allows for adipose fat mobilization in early lactation (Bell, 1995). Thus, heat-stressed cows have less lipid mobilization from adipose tissue and fewer ketone bodies available to use for energy relative to thermoneutral cows. Pair-feeding studies have also demonstrated alterations in glucose metabolism associated with heat stress. Lactating heat-stressed cows had faster glucose clearance from systemic circulation following exogenous glucose administration (Wheelock et al., 2010). Furthermore, plasma glucose appearance after glucose infusion, on a milk yield basis, was approximately 6% higher in heat-stressed relative to pair-fed thermoneutral cows (Baumgard et al., 2011). Along with the higher circulating insulin concentrations and greater glucose uptake in lactating heat-stressed cows, it is apparent that thermal stress increases peripheral glucose use (Wheelock et al., 2010). Thus, lactating heat-stressed cows have a reduced capacity to initiate the glucose sparing mechanisms and NEFA mobilization necessary to partition resources to the mammary gland for milk synthesis. Of note, cows that are heat stressed only during the dry period, and subsequently cooled after calving, experience similar alterations in glucose and lipid metabolism as lactating heat-stressed cows (do Amaral et al., 2009; Tao et al., 2012). Thus, heat stress during the dry period can have carry-over effects on the metabolic physiology of the cow during the subsequent lactation.

Mitochondria are important stress sensors and responders that modulate the energetic response to stress, making them pivotal organelles for stress adaptation (Picard et al., 2018). All aspects of the cellular and systemic response to stress require energy, a demand that is largely met by increased mitochondrial respiration (Picard et al., 2018). Cortisol and catecholamines stimulate mobilization of substrates from body stores and enhance gluconeogenesis, providing substrates and oxygen for ATP synthesis in critical tissues during stress (Charmandari et al., 2005). Glucocorticoid and catecholamine (i.e., epinephrine, norepinephrine) signaling also induces changes in mitochondrial structure as well as mitochondrial biogenesis and respiration across cell and tissue types (Picard et al., 2018).

Although there is evidence of elevated cortisol and epinephrine concentrations with heat stress in dairy cows (Alvarez and Johnson, 1973; Titto et al., 2017), the mitochondrial response to heat stress has rarely been studied, particularly within the context of energy production during lactation. We recently conducted a study to assess the effects of heat stress on mitochondrial structure and function in lactating, heat-stressed cows. Multiparous Holstein cows in mid lactation were assigned to either a heat-stressed group (fitted with electric heat blankets) or a pair-fed thermoneutral group (held at thermoneutral conditions and pair-fed to match the feed intake reduction in the heat-stressed group) for 10 d following a 7-d baseline period when all cows were thermoneutral. Although respiration rates and rectal temperatures were elevated in the heat-stressed group, the values were lower than previous reports of moderate heat stress in dairy cows, indicating that in our study, a mild heat stress was induced. Similar to previous observations, milk yield and NEFA concentrations in our study dropped in the heat-stressed group when wearing the blankets relative to baseline. However, there was no measurable impact of heat stress on mitochondrial RCR, oxidant emission, or oxidative damage in the liver. Not surprisingly, there were also no differences between treatment groups in the expression of genes involved in mito-nuclear signaling pathways, including *PPARGC1A*, *NRF*, or *TFAM*/*TFBM* (Marquez-Acevedo et al., 2022). However, there were fewer mitochondria in mammary tissue sections from the heat-stressed relative to the pair-fed thermoneutral group (Marquez-Acevedo et al., 2023a). Our results indicate that hepatic mitochondria are able to cope with mild heat stress, and that altered mammary mitochondrial biogenesis may contribute somewhat to the reduction in milk yield in the heat-stressed cows. It is also plausible that higher thermal loads may surpass mitochondrial limits for adaptation to changing environmental conditions, as demonstrated with exogenous glucocorticoid administration and with other stressors (Duclos et al., 2004; García-Roche et al., 2019).

Adaptive physiological responses to heat stress in dairy cows are regulated at the molecular level by changes in the expression of metabolic genes and proteins, many of which are involved in biochemical processes occurring within mitochondria. In the liver, downregulation of genes such as *CPT1A*, *ACADVL*, *PPARA*, *SCD*, *FADS2*, and *APOB* may underlie reduced hepatic β-oxidation of FA, depressed FA synthesis, and reduced low density lipoprotein synthesis in heat-stressed cows (do Amaral et al., 2009; Shahzad et al., 2015). Enhanced hepatic gluconeogenesis in heat-stressed cows is indicated by upregulation of propanoate and butanoate metabolic pathways and the pyruvate carboxylase gene, encoding a rate-limiting enzyme that catalyzes the carboxylation of pyruvate to form oxaloacetate in the mitochondrially localized first step of the gluconeogenic pathway (Shahzad et al., 2015). We conducted a proteomics analysis of liver samples from heat-stressed cows to characterize changes in hepatic protein expression with heat stress, with a particular interest in metabolic proteins. In this study, liver samples were taken at 2 d relative to parturition from cows that were either cooled (i.e., had access to water soakers and fans) or moderately heat stressed (i.e., lacked access to cooling devices) during the entire dry period (∼46 d) but were cooled postpartum. The top 2 functional pathways of proteins differentially expressed between treatment groups were mitochondrial dysfunction and OXPHOS. Several proteins in these pathways included core and accessory subunits of complex I and IV of the ETC and proteins involved in oxidant defense, which were all downregulated in the heat-stressed group relative to the cooled group, suggestive of impediments to OXPHOS, ATP production, and oxidant neutralization in the liver of heat-stressed cows (Skibiel et al., 2018). Similarly, a proteomics analysis of liver tissue from cows heat stressed in mid lactation also revealed lower abundances of several OXPHOS proteins relative to thermoneutral cows (Ma et al., 2019). Moderate thermal loads appear to downregulate numerous hepatic genes and proteins involved in mitochondrial metabolism, which may contribute to oxidative stress, energy deficits, and impaired ability of heat-stressed cows to partition nutrients for milk synthesis.

This review exemplifies the crucial role of hepatic mitochondria in the biochemical and metabolic processes occurring in the lactating cow that enables copious milk production across lactation in accordance with temporal variation in energetic and nutrient requirements. Coordinated changes in mitochondrial density and substrate use for respiration in the liver may be important adaptations to the energy demands of lactation and appear to be mediated by signaling pathways involved in regulating mitochondrial biogenesis and function along with hepatic macronutrient metabolism. However, molecular pathways involved in regulating mitochondrial metabolism across lactation in the liver and other tissues and are still poorly understood. Available evidence from “omics” and traditional studies also suggests that mitochondrial dysfunction in the liver, and likely other tissues, is a consequence of heat stress that may impede cattle health and lactation performance. Further research is needed to characterize cell signaling pathways contributing to mitochondrial homeorhesis across life and productive stages as well as in response to stress. Additionally, exploring pathways communicating metabolic status across tissues in the context of mitochondrial function during lactation would shed light on how tissue-specific mitochondrial adjustments are modulated across the body to influence systemic metabolism. With this knowledge, it may be possible to develop more advanced nutritional programs for lactating cows that facilitate achievement of genetic potential for milk production and aid in the development of better management strategies and pharmaceutical approaches to combat metabolic disorders and health and production issues stemming from thermal stress.
